# Management of Facial Nerve Paresis After Nerve-Preserving Parotidectomy: A Review

**DOI:** 10.3390/jcm15218417

**Published:** 2026-10-29

**Authors:** Fabio Maglitto, Giovanni Salzano, Serena Trotta, Eutilia Manzo, Valentino Vellone, Umberto Committeri, Luigi Angelo Vaira, Giovanni Dell’Aversana Orabona

**Affiliations:** 1 Maxillofacial Surgery Unit, Department of Neurosciences, Reproductive and Odontostomatological Sciences, University of Naples Federico II, 80131 Naples, Italy; fabio.maglitto@aoufederico2.unina.it (F.M.); giovannisalzanomd@gmail.com (G.S.); serenatrotta99@icloud.com (S.T.); giovanni.dellaversanaorabona@unina.it (G.D.O.); 2 Department of Life Science, Health and Health Professions, Università Degli Studi “Link”, 00165 Rome, Italy; v.vellone@unilink.it; 3 Department of Maxillofacial Surgery, “S. Maria” Hospital, 05100 Terni, Italy; umbertocommitteri@gmail.com; 4 Maxillofacial Surgery Unit, University Hospital of Sassari, 07100 Sassari, Italy; lavaira@uniss.it

**Keywords:** parotidectomy, facial nerve paresis, facial nerve palsy, facial rehabilitation, corticosteroids, neuromuscular retraining, scoping review

## Abstract

**Background/Objectives:** Postoperative facial nerve paresis remains clinically relevant after nerve-preserving parotidectomy. This scoping review aimed to identify, map, and characterize reported therapeutic strategies and summarize clinical recovery outcomes. **Methods:** Following Joanna Briggs Institute guidance and PRISMA-ScR, the protocol was deposited on Figshare before data extraction. PubMed, Embase, and Scopus were searched through 24 August 2026. English-language original studies of treatments initiated after the recognition of paresis were eligible. Two reviewers independently selected and appraised studies; one reviewer charted the data and another verified the extraction. **Results:** Eight studies involving 211 patients were included; reported ages were 17–86 years. Procedures included partial, superficial, subtotal, total, and revision parotidectomy, as well as parotid lobectomy for benign or malignant disease. Immediate or early weakness predominated; two reports described delayed-onset palsy. Evidence comprised two randomized trials, one case–control study, one multicenter cohort, and four case reports. Interventions included corticosteroids, corticosteroid–antiviral therapy, home or supervised exercises, Kabat rehabilitation, neuromuscular retraining, Matrix Rhythm Therapy (MRT), and proprioceptive neuromuscular facilitation with electrical stimulation. The trials found no benefit of prednisolone over placebo and no statistically significant difference between supervised rehabilitation and home exercises; equivalence was not established. Observational findings could not be separated from spontaneous recovery or co-treatment. **Conclusions:** No intervention has established superiority. Standardized protocols, validated outcomes, and adequately powered comparative studies are needed.

## 1. Introduction

Parotidectomy is the principal surgical treatment for a broad range of benign and malignant parotid gland neoplasms. Because the facial nerve traverses the parotid gland and divides into its terminal branches within the glandular parenchyma, preservation of its anatomical and functional integrity represents a central objective of parotid surgery [1,2]. Even when the anatomical continuity of the facial nerve is maintained, postoperative weakness may result from traction, manipulation, compression, thermal injury, ischemia or postoperative edema. These mechanisms may produce neurapraxic or axonotmetic injury without visible nerve transection [3,4]. Consequently, most patients with an anatomically preserved nerve experience partial or complete spontaneous recovery, although the trajectory may extend over several months and can vary according to the initial severity and underlying mechanism of injury [4,5]. Delayed-onset facial palsy occurring after initially preserved postoperative function represents a less common clinical presentation and may involve inflammatory or viral-reactivation mechanisms [6,7]. Despite its usually transient nature, postoperative facial paresis can cause meaningful morbidity during the recovery period. Impaired eyelid closure may compromise ocular protection, while weakness of the oral and perioral musculature can interfere with speech, oral competence, eating and drinking. Facial asymmetry and reduced facial expressiveness may also negatively affect social interaction, emotional well-being and health-related quality of life [8]. Accelerating recovery, limiting secondary complications and supporting patients throughout the recovery process are therefore clinically relevant objectives even when the long-term prognosis is favorable. Several therapeutic approaches have been proposed for postoperative facial nerve paresis following nerve-preserving parotidectomy. Pharmacological management has primarily involved corticosteroids, sometimes combined with antiviral agents in cases of delayed-onset paralysis. However, a systematic review of two randomized trials found no evidence that corticosteroid treatment improved facial nerve recovery compared with no corticosteroid treatment [3]. Rehabilitation strategies reported in this population include home-based facial exercises, mirror-assisted exercises, massage, supervised facial rehabilitation, neuromuscular retraining, proprioceptive neuromuscular facilitation, electrical stimulation and, more recently, adjunctive modalities such as Matrix Rhythm Therapy (MRT) [4,5,9,10,11]. The available evidence is difficult to interpret because the literature encompasses randomized trials, observational studies and individual case reports, with substantial variation in treatment protocols, timing of intervention, duration of follow-up and outcome measurement. Facial recovery has been evaluated using clinician-rated instruments—including the House–Brackmann grading system and the Sunnybrook Facial Grading System [12,13]—as well as regional assessments, electrophysiological measures and patient-reported outcomes. Moreover, existing evidence syntheses have examined corticosteroid therapy or physical therapy for iatrogenic facial paralysis separately, rather than mapping the full range of therapeutic strategies specifically used after nerve-preserving parotidectomy [3,11].

A scoping review is therefore appropriate to identify the extent and characteristics of this heterogeneous evidence base, describe how postoperative facial nerve recovery has been assessed and highlight areas in which evidence remains limited or inconsistent. The contribution of this review is to map pharmacological and rehabilitation strategies within the same postoperative population, explicitly separating treatment of an established deficit from perioperative prevention. It also maps treatment timing, outcome instruments and the limitations that prevent causal attribution of recovery.

### 1.1. Aim

This scoping review aimed to identify, map and characterize the therapeutic strategies reported for the management of postoperative facial nerve paresis following nerve-preserving parotidectomy and to summarize the documented clinical recovery outcomes.

### 1.2. Review Question

What therapeutic strategies have been reported for the management of postoperative facial nerve paresis following nerve-preserving parotidectomy, and what recovery outcomes have been documented?

## 2. Materials and Methods

### 2.1. Review Design and Protocol

This scoping review was conducted in accordance with the Joanna Briggs Institute (JBI) guidance for scoping reviews [14] and is reported according to PRISMA-ScR [15]. The protocol was deposited on Figshare on 24 August 2026, before data extraction and completion of the review (https://doi.org/10.6084/m9.figshare.33322038). The database searches were also conducted on 24 August 2026.

#### 2.1.1. Eligibility Criteria

Eligibility criteria were defined according to the Population–Concept–Context framework. The population comprised patients of any age who developed postoperative facial nerve paresis or paralysis following parotidectomy in which the anatomical continuity of the facial nerve was preserved. Studies involving deliberate or accidental facial nerve sacrifice, transection, grafting or reconstruction were excluded. Studies including patients with pre-existing facial nerve dysfunction or mixed surgical populations were considered eligible only when data relating specifically to new-onset postoperative dysfunction after nerve-preserving parotidectomy could be identified separately. As specified in Supplementary Material S2, the review also retained reports in which nerve continuity was inferred from the operative and recovery course without reported sacrifice or reconstruction. Such inferred continuity was distinguished from explicit operative confirmation and treated as an eligibility-related limitation, rather than proof of anatomical preservation.

The concept of interest was the therapeutic management of established postoperative facial nerve dysfunction. Eligible interventions included pharmacological treatment, structured facial exercise therapy, neuromuscular retraining, proprioceptive neuromuscular facilitation, massage, electrical stimulation, other physical modalities and multimodal rehabilitation programs. Interventions had to be initiated after the onset or clinical recognition of postoperative facial nerve dysfunction. Studies evaluating exclusively preventive or perioperative interventions administered before postoperative paresis was established were excluded. Studies limited to incidence, risk factors, prognostic factors, surgical technique, intraoperative nerve monitoring, natural history or recovery without an identifiable post-onset therapeutic strategy were also excluded.

The context included partial, superficial, subtotal, total or revision parotidectomy undertaken for benign or malignant parotid disease in any inpatient, outpatient, rehabilitation or home-based setting. Studies involving facial nerve palsy from other causes were excluded unless findings for the post-parotidectomy population were reported separately.

Original full-text journal articles published in English were eligible, including randomized and nonrandomized trials, cohort studies, case–control studies, case series and case reports. No publication-date restriction was applied. Reviews, systematic reviews, guidelines, editorials, letters without original clinical data, conference abstracts, theses, dissertations, preprints and other gray literature were excluded. Eligible studies were required to report at least one post-treatment clinical outcome relating to facial nerve recovery, severity of dysfunction, time to recovery, residual deficit, synkinesis, functional status, patient-reported outcome or treatment safety.

#### 2.1.2. Search Methods

A comprehensive literature search was conducted in PubMed, Embase and Scopus on 24 August 2026. The search strategy combined controlled vocabulary, where available, and free-text terms relating to parotidectomy and facial nerve dysfunction. Terms describing the surgical context included parotidectomy, parotid surgery, parotid gland surgery and parotid resection. Terms describing facial nerve dysfunction included facial nerve paresis, facial nerve palsy, facial paralysis, facial weakness, facial nerve dysfunction, facial nerve injury, facial nerve deficit, facial nerve recovery and facial nerve outcomes. The PubMed strategy was developed first and subsequently adapted to the syntax and indexing systems of Embase and Scopus. No publication-date restriction was applied. Eligibility was restricted to English-language full texts. The saved PubMed and Embase strategies contain English-language limits; the saved Scopus string does not contain a language-limit clause. English-language eligibility was applied during study selection. The complete reproducible search strategies for all databases are provided in Supplementary Material S1.

All retrieved records were imported into EndNote 2025 (Clarivate, Philadelphia, PA, USA). Duplicate records were identified and removed using the automated duplicate-detection function in EndNote. The resulting duplicate groups and potentially ambiguous matches were subsequently checked manually by the review team before screening. The numbers of records retrieved from each database and removed as duplicates were recorded for inclusion in the PRISMA flow diagram.

#### 2.1.3. Study Selection Process

The database searches identified 4425 records. Before formal screening, the reviewers discussed the eligibility criteria and applied them to a pilot set of records to ensure a consistent interpretation. Two reviewers independently screened the titles and abstracts of all retrieved records against the predefined eligibility criteria. Records considered potentially eligible by either reviewer, or for which eligibility could not be determined from the title and abstract, were retained for full-text assessment. The same two reviewers independently assessed the full texts of potentially eligible reports. Eligibility was determined with particular attention to anatomical preservation of the facial nerve, initiation of a therapeutic or rehabilitative strategy after the onset of postoperative facial nerve dysfunction and availability of extractable clinical recovery outcomes. Disagreements at either stage were resolved through discussion and consensus between the two reviewers. Reasons for exclusion at the full-text stage were documented. The study-selection process was summarized using a PRISMA flow diagram.

#### 2.1.4. Data Charting

A standardized data-charting form was developed specifically for this review and piloted before final extraction. The form was refined iteratively to ensure that it captured the clinical and methodological heterogeneity of the included evidence. Data were charted from each included report by one reviewer and independently verified against the corresponding full text by a second reviewer. Discrepancies or uncertainties were resolved through discussion and consensus, with direct verification of the source article. No study authors were contacted for additional or missing information. When information was not reported or could not be determined reliably, it was recorded as “not reported” rather than inferred.

#### 2.1.5. Data Items

The following information was charted: bibliographic characteristics, including first author, publication year, country and journal; study design and clinical setting; sample size and participant characteristics; indication for parotidectomy and tumor histology; type and extent of parotidectomy; confirmation of facial nerve preservation; timing, severity and pattern of postoperative facial nerve dysfunction; therapeutic strategy, treatment components, dosage or intensity, timing of initiation and treatment duration; comparator or concurrent treatment, where applicable; facial nerve assessment instruments; duration and timing of follow-up; facial nerve recovery, including changes in severity, complete or incomplete recovery and time to recovery; residual deficits, synkinesis, functional outcomes and patient-reported outcomes; adverse events and treatment tolerability; authors’ principal conclusions and reported study limitations.

Where studies used different facial nerve grading systems, outcomes were charted as originally reported. Scores from different instruments were not converted into a common scale because such conversions have not been consistently validated.

#### 2.1.6. Collation, Summarization and Reporting of Results

The characteristics of the included studies were summarized in evidence tables and described narratively. Therapeutic strategies were grouped to match the Results subsections: pharmacological management; facial exercise therapy and supervised rehabilitation; Kabat rehabilitation and proprioceptive neuromuscular facilitation; facial neuromuscular retraining; and MRT and multimodal physical therapy. Recovery outcomes were mapped according to the instruments used, direction and magnitude of reported change, recovery time, proportion achieving complete recovery, and presence of persistent or residual dysfunction. Particular attention was given to treatment initiation, baseline severity, treatment duration, and follow-up. A quantitative meta-analysis was not undertaken because the objective was to map the evidence and substantial heterogeneity was present across study designs, interventions, comparator groups, outcome instruments, and assessment time points. Findings were therefore synthesized descriptively without calculating a pooled treatment effect.

#### 2.1.7. Critical Appraisal of Individual Sources of Evidence

Although critical appraisal is not mandatory for scoping reviews, a methodological-quality assessment was undertaken to support the interpretation of the reported therapeutic and recovery outcomes. Two reviewers independently appraised each included study using the Joanna Briggs Institute critical appraisal checklist appropriate to its design. The JBI checklists for randomized controlled trials, case–control studies, cohort studies and case reports were applied as appropriate. Each checklist item was rated as “yes”, “no”, “unclear” or “not applicable”. Disagreements between the reviewers were resolved through discussion and consensus. Studies were not excluded on the basis of methodological quality because the purpose of the review was to map the extent and characteristics of the available evidence. Appraisal findings were instead used to contextualize the certainty and interpretability of the reported outcomes, particularly with regard to uncontrolled study designs, spontaneous facial nerve recovery, concurrent interventions and incomplete outcome reporting.

#### 2.1.8. Patient and Public Involvement

Patients and members of the public were not involved in the design, conduct or reporting of this scoping review.

## 3. Results

### 3.1. Study Selection

The database searches identified 4425 records, comprising 1157 records from PubMed, 1512 from Embase and 1756 from Scopus. After the removal of 2200 duplicates, 2225 unique records underwent title and abstract screening. Of these, 2214 records were excluded because they did not address the management of postoperative facial nerve dysfunction following nerve-preserving parotidectomy.

**Figure 1:**
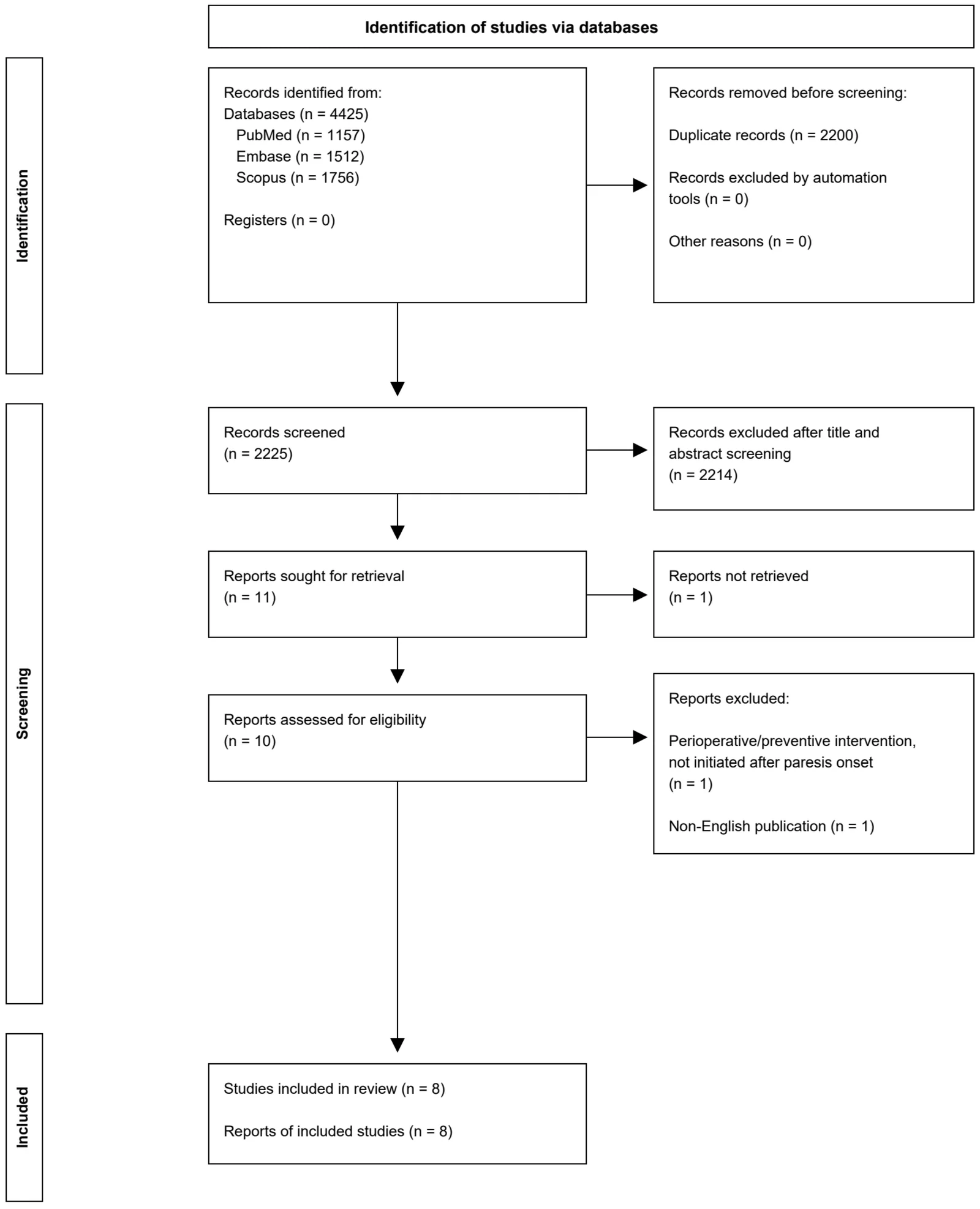
**PRISMA flow diagram of study identification and selection.** Searches of PubMed, Embase and Scopus identified 4425 records. After removal of 2200 duplicates, 2225 records underwent title and abstract screening. Eleven reports were sought for retrieval, one could not be retrieved, 10 were assessed in full text, two were excluded with reasons, and eight studies were included in the scoping review.

Eleven reports were sought for retrieval. One report could not be retrieved, leaving 10 reports for full-text assessment. Two reports were excluded after full-text evaluation: one evaluated a perioperative preventive intervention initiated before postoperative facial nerve paresis was established, and one was published in a language other than English. Eight studies fulfilled the eligibility criteria and were included in the scoping review. The study-selection process is presented in Figure 1.

### 3.2. Study Characteristics

The eight included studies were published between 2008 and 2026 and were conducted in seven countries: the Republic of Korea, Spain, Italy, the United States, India, Japan and Iraq [4,5,6,7,9,10,16,17]. Two studies were conducted in Italy. The evidence base comprised two randomized controlled trials [5,16], one retrospective case–control study [17], one retrospective multicenter cohort study [4] and four case reports [6,7,9,10].

The charted therapeutic-outcome populations comprised 211 patients with postoperative facial nerve paresis (44 + 61 + 56 + 46 + four individual cases). This total uses the 44 evaluable patients in the prednisolone trial and the 61 patients with paresis in the facial-exercise trial, rather than all randomized participants. Study sample sizes varied markedly, ranging from individual case reports to a randomized study involving 79 participants, 61 of whom developed postoperative facial paresis. The studies included patients undergoing superficial, partial, subtotal or total parotidectomy. Extracapsular dissection, parotid lobectomy and revision parotid surgery were also represented. Both benign and malignant parotid neoplasms were included.

Documentation of nerve preservation varied. Six studies described preservation through operative information, exclusion of sacrifice or transection, intraoperative stimulation or classification as nerve-sparing surgery [4,5,6,7,16,17]. In the Dharwadkar and Alwash case reports, continuity was inferred in the original charting from the clinical course rather than explicitly documented in the extracted operative information [9,10]. These two reports therefore provide less secure evidence for the strictly nerve-preserving population (Table S1).

The timing and severity of facial nerve dysfunction varied across studies. Most studies evaluated weakness occurring in the immediate postoperative period or within the first postoperative week [4,5,16,17]. Two reports described delayed severe paralysis at postoperative days 12 and 16 [6,7]; the latter patient had an initial mild lower-lip deficit (HB II), followed by worsening to HB V. Baseline severity ranged from mild regional weakness to complete facial paralysis. The House–Brackmann grading system was the most frequently used outcome instrument and was reported in all eight studies. Additional assessment methods included the Sunnybrook Facial Grading System, Facial Disability Index, facial manual muscle testing, strength–duration curves, electroneurography, the Synkinesis Assessment Questionnaire and the International Classification of Functioning, Disability and Health framework.

The principal characteristics of the eight included studies—including study design, geographical setting, sample characteristics, surgical context, baseline facial nerve dysfunction, outcome measures and follow-up duration—are summarized in Table 1.

### 3.3. Therapeutic Strategies and Recovery Outcomes

The therapeutic strategies identified in the included studies comprised pharmacological treatment, structured facial exercise therapy, neuromuscular retraining, proprioceptive neuromuscular facilitation and multimodal physical therapy. Considerable variation was observed in the timing, duration and intensity of treatment. Interventions were initiated between the first postoperative day and 37 days after surgery and lasted from 10 days to more than 12 months.

Three studies described primarily pharmacological management [6,7,16], whereas five investigated rehabilitation or physical therapy interventions [4,5,9,10,17]. Only three studies included a comparator group [5,16,17]. The remaining evidence consisted of one uncontrolled cohort and four individual case reports.

The therapeutic strategies, treatment protocols, timing and duration of treatment, comparators, reported recovery outcomes, safety findings and evidence-specific interpretations are summarized in Table 2.

#### 3.3.1. Pharmacological Management

Roh and Park conducted a randomized, double-blind, placebo-controlled trial evaluating oral prednisolone in patients who developed facial nerve palsy after anatomically nerve-preserving parotidectomy [16]. Prednisolone was initiated on the first or second postoperative day and administered for 10 days using a tapering regimen of 50 mg/day for 5 days, 30 mg/day for 3 days and 10 mg/day for 2 days. Twenty-two evaluable patients received prednisolone and 22 received placebo. Complete facial nerve recovery occurred in 37 of 44 patients (84%) within 3 months and 43 of 44 patients (98%) within 6 months. Recovery rates did not differ significantly between the prednisolone and placebo groups at any assessment point. One patient who had undergone total parotidectomy had persistent facial nerve dysfunction at 18 months. The trial therefore found no evidence that postoperative prednisolone accelerated recovery or increased the probability of complete recovery compared with placebo. Two case reports described corticosteroid treatment for delayed-onset facial nerve paralysis [6,7]. Ostrowski et al. reported a patient with normal immediate postoperative facial nerve function who developed House–Brackmann grade VI paralysis 12 days after total parotidectomy [6]. The patient received prednisone 20 mg three times daily for 7 days followed by a 3-day taper. A second course was administered after partial improvement. Facial nerve function improved to House–Brackmann grade II by 10 weeks. Kuba et al. reported severe delayed facial palsy developing 16 days after nerve-preserving parotid surgery [7]. After exclusion of evident postoperative infection, hematoma, edema and tumor recurrence, the patient received intravenous prednisolone sodium succinate, initially at 100 mg/day and tapered to 40 mg/day over 1 week, followed by oral corticosteroid tapering. Intravenous acyclovir at 5 mg/kg every 8 h was administered concurrently for 1 week because viral reactivation was considered a possible mechanism. Complete facial nerve recovery was documented 4 months after onset, with no recurrence during more than 1 year of surveillance. Both delayed-onset cases improved after treatment. However, the absence of comparator groups and the favorable natural history of facial nerve dysfunction with preserved anatomical continuity prevented attribution of recovery specifically to corticosteroids or antiviral treatment.

#### 3.3.2. Facial Exercise Therapy and Supervised Rehabilitation

Infante-Cossio et al. compared a home-based facial exercise program with supervised rehabilitation following conventional superficial parotidectomy [5]. Participants allocated to the home program received instructions for 10 facial movements performed in front of a mirror five times daily. In the supervised rehabilitation group, patients with moderate-to-severe paresis received 10 weekly 45-min sessions comprising supervised facial exercises, external and intraoral massage, technique correction and daily home exercises. Patients with only slight paresis performed self-massage and mirror exercises without additional supervised sessions. No statistically significant differences were found between the groups in the prevalence, magnitude, duration or probability of recovery of facial paresis at any follow-up point. Complete facial mobility was recovered within 12 months in the absence of intraoperative nerve injury, irrespective of treatment allocation. The trial did not detect a statistically significant between-group difference; this should not be interpreted as proof of equivalence or non-inferiority. These findings did not demonstrate an additional recovery benefit from the more resource-intensive supervised program.

#### 3.3.3. Kabat Rehabilitation and Proprioceptive Neuromuscular Facilitation

Boschetti et al. evaluated early Kabat rehabilitation in patients with severe postoperative facial nerve palsy, defined as House–Brackmann grade IV or V at postoperative day 7 [17]. Kabat rehabilitation commenced on postoperative day 7 and was administered three times weekly for 12–24 weeks. The protocol used diagonal and spiral proprioceptive neuromuscular facilitation movements targeting upper, intermediate and lower facial muscle groups. At 3 months, the mean House–Brackmann grade was 0.71 grades lower in the Kabat group than in the non-Kabat group. The probability of remaining in a higher House–Brackmann grade was reduced by approximately 13% among patients receiving Kabat rehabilitation. Among patients who initially presented with House–Brackmann grade V palsy, 45% of those receiving Kabat rehabilitation had reached grade I at 3 months, compared with none of the controls. The study suggested faster short-term improvement with Kabat rehabilitation, particularly among patients with more severe initial dysfunction. Nevertheless, the retrospective and nonrandomized design left the findings vulnerable to selection bias and residual confounding. Alwash reported proprioceptive neuromuscular facilitation combined with faradic electrical stimulation in a patient with House–Brackmann grade IV palsy following total parotidectomy [10]. Treatment began approximately 4 weeks after surgery and comprised 12 sessions over 6 weeks. Proprioceptive neuromuscular facilitation was delivered for approximately 20–30 min per session, while faradic stimulation was applied for 20 min at 50 Hz. Daily home exercises were also prescribed. After 6 weeks, facial symmetry, eye closure and facial expression had improved, with the House–Brackmann grade decreasing from IV to II. The Facial Disability Index physical function score increased from 44 to 93, while the social and well-being score increased from 64 to 94. Improvement was maintained at 1 year, although mild residual weakness of the platysma persisted. Because proprioceptive neuromuscular facilitation, electrical stimulation and home exercises were delivered concurrently, the contribution of each treatment component could not be determined.

#### 3.3.4. Facial Neuromuscular Retraining

Molinari et al. evaluated facial neuromuscular retraining in 46 patients who developed facial palsy following nerve-sparing partial, subtotal, total or revision parotid surgery [4]. Rehabilitation began a mean of 15 days after surgery and consisted of selective small, slow and symmetrical facial movements, facial massage, mirror feedback and daily home practice. The program was initially taught by a dedicated speech therapist and subsequently performed independently by patients. At the initial rehabilitation assessment, the mean Sunnybrook score was 54, and 18 patients (39.1%) had House–Brackmann grade IV–VI dysfunction. The mean Sunnybrook score increased to 76.5 at 2 months and 95.4 at 6 months. Complete recovery was reported in 72% of patients at 12 months, 85% at 18 months and 89.1% at 24 months. No patient continued to have House–Brackmann grade IV–VI dysfunction after 12 months; however, five patients had persistent grade II weakness at 24 months. The greatest improvement occurred during the first 6 months, although further recovery was observed through 18 months. The absence of an untreated comparator meant that the contribution of neuromuscular retraining could not be separated from spontaneous recovery, concurrent corticosteroid treatment or other prognostic factors.

#### 3.3.5. MRT and Multimodal Physical Therapy

Dharwadkar et al. described MRT as an adjunct to conventional physiotherapy in a patient with House–Brackmann grade IV palsy after superficial parotidectomy [9]. MRT was applied for 45 min once weekly over 3 consecutive weeks to the cervical region and facial musculature. Concurrent treatment included facial exercises, mirror therapy, proprioceptive neuromuscular facilitation and functional facial exercises performed three times daily. Following treatment, facial nerve function improved from House–Brackmann grade IV to grade II. Manual muscle-testing scores for the frontalis, orbicularis oculi and orbicularis oris increased from 2 to 4, accompanied by improved eye closure, smiling and pouting. Changes were also documented in the International Classification of Functioning, Disability and Health profile. As MRT was administered with several other rehabilitation components, its independent therapeutic contribution could not be established.

### 3.4. Critical Appraisal of Included Studies

Methodological quality varied considerably according to study design. No study was excluded on the basis of the critical appraisal because the purpose of the review was to map the available evidence. Among the two randomized controlled trials, Infante-Cossio et al. reported computer-generated randomization, allocation concealment using sealed masked envelopes, blinded outcome assessment and an intention-to-treat analysis [5]. Participants and treating clinicians could not be blinded because of the nature of the rehabilitation interventions. Roh and Park described their study as a randomized, double-blind, placebo-controlled trial and used blinded assessment by two independent evaluators [16]. However, the methods used to generate the randomization sequence and conceal treatment allocation were not sufficiently described. In addition, one participant assigned to prednisolone was lost to follow-up and excluded from the final analysis. The retrospective case–control study by Boschetti et al. applied consistent eligibility criteria and outcome assessments to both groups and used regression and difference-in-differences analyses to account for measured prognostic variables [17]. Nevertheless, patients were not individually matched, and participation in Kabat rehabilitation was not randomly assigned. Residual confounding and selection bias therefore remained possible. The multicenter cohort study by Molinari et al. used validated facial nerve assessment instruments, repeated evaluations and multivariable analyses [4]. Its principal limitations were the absence of an untreated comparator, variability in available follow-up and the lack of a specific statistical strategy for incomplete longitudinal observations. Consequently, improvements documented during neuromuscular retraining could not be separated definitively from spontaneous facial nerve recovery or concurrent medical treatment. The four case reports generally provided clear descriptions of the patients, clinical presentation, therapeutic interventions and post-treatment outcomes [6,7,9,10]. Alwash additionally reported treatment adherence, long-term follow-up and the absence of adverse events, skin irritation, muscle spasms or treatment-induced synkinesis [10]. The delayed-palsy reports did not explicitly assess or report treatment-related adverse events [6,7]. The MRT case report explicitly stated that no side effects or complications were observed after treatment, although the method of adverse-event assessment was not described [9]. Because these publications lacked comparator groups, they established temporal associations between treatment and improvement but could not demonstrate therapeutic efficacy or exclude spontaneous recovery. Overall, the two randomized trials provided the most informative comparative evidence, although they investigated different interventions and therefore could not be quantitatively combined. Evidence supporting the remaining therapeutic strategies was derived predominantly from observational studies and individual case reports and should consequently be interpreted as exploratory. The results of the design-specific JBI critical appraisal are presented in Table 3. Appraisal findings were used to contextualize the strength and limitations of the available evidence and were not used as eligibility criteria.

### 3.5. Patterns and Timing of Facial Nerve Recovery

Recovery trajectories varied according to baseline severity, surgical context and duration of follow-up. In the randomized prednisolone trial, 84% of evaluable patients recovered completely within 3 months and 98% within 6 months, irrespective of treatment allocation [16]. In the facial exercise trial, complete recovery occurred within 12 months in both intervention groups [5]. The observational rehabilitation studies suggested that the most pronounced improvement occurred within the first 3–6 months [4,17]. Nevertheless, Molinari et al. documented additional recovery between 12 and 18 months, indicating that improvement may continue beyond the first postoperative year in patients with incomplete early recovery [4]. The two delayed-onset cases achieved complete or near-complete recovery within approximately 10 weeks and 4 months, respectively [6,7]. The multimodal rehabilitation case reports demonstrated improvements over shorter treatment periods of 3–6 weeks, although residual grade II weakness remained in some facial regions [9,10]. Overall, complete or near-complete recovery was common when the anatomical continuity of the facial nerve had been preserved. However, recovery time varied substantially, and patients with more severe initial dysfunction could experience residual weakness or require prolonged rehabilitation.

### 3.6. Safety and Adverse Events

Safety outcomes were inconsistently reported. In the prednisolone trial, one patient experienced minor steroid-related symptoms, while no major treatment-related adverse events were observed [16]. The electrical stimulation and proprioceptive neuromuscular facilitation case report explicitly documented the absence of adverse events, skin irritation, muscle spasms and treatment-induced synkinesis [10]. For the facial exercise, Kabat and neuromuscular retraining studies, the absence of described harms should not be equated with systematic demonstration of safety [4,5,17]. The MRT case report stated that no side effects or complications were observed after treatment [9]. Adverse-event collection methods were generally not specified. Similarly, the two reports of corticosteroid treatment for delayed palsy did not provide a systematic assessment of treatment-related adverse effects [6,7]. The available evidence was therefore insufficient to compare the safety profiles of different therapeutic strategies.

### 3.7. Evidence Gaps

The evidence base was limited by the small number of comparative studies, marked variation in intervention protocols and inconsistent outcome reporting. Only two randomized trials were identified, and these evaluated different interventions [5,16]. The remaining evidence consisted of observational studies and individual case reports. The House–Brackmann system was used consistently, but additional outcome instruments and assessment schedules varied. Patient-reported outcomes were evaluated in only one study [10], while quality of life, oral competence, ocular symptoms, social participation and treatment satisfaction were rarely assessed systematically. Adverse-event reporting was also incomplete. No comparative evidence was identified for electrical stimulation, MRT, antiviral therapy or long-term neuromuscular retraining. Furthermore, the available studies did not consistently distinguish the effects of treatment from spontaneous recovery or concurrent therapies. Standardized intervention protocols, common outcome measures and adequately controlled prospective studies are therefore required to determine whether specific therapeutic strategies accelerate recovery or improve its final extent.

## 4. Discussion

This scoping review identified eight methodologically heterogeneous studies: two randomized controlled trials, one retrospective case–control study, one retrospective multicenter cohort study, and four case reports. Comparative evidence was scarce, and most interventions were supported only by observational or single-patient evidence. Recovery was generally favorable when facial nerve continuity had been preserved, although it ranged from a few weeks to more than 1 year [4,5,6,7,9,10,16,17]. The recovery rates in the included comparative studies were broadly consistent with, rather than clearly superior to, natural-history data. In the prednisolone trial, complete recovery across treatment groups reached 84% at 3 months and 98% at 6 months [16]; the facial-exercise trial reported complete recovery within 12 months in both groups [5]. In a separate natural-history cohort of immediate postoperative dysfunction, Tung et al. reported cumulative recovery rates of 31%, 70%, 81%, and 83% at 1, 3, 6, and 12 months, respectively, and initial dysfunction greater than House–Brackmann grade III was the main adverse prognostic factor [18]. These cross-study comparisons cannot establish treatment benefit because baseline severity, surgical extent, outcome definitions, follow-up, and rehabilitation exposure differed. Molinari et al. also observed further improvement through 18 months in some patients receiving neuromuscular retraining [4], supporting longer follow-up for substantial or incomplete early recovery. Even transient weakness can impair eyelid closure, oral competence, speech, and emotional expression. After superficial parotidectomy, the greatest reduction in Facial Disability Index scores coincided with peak weakness during the first postoperative week, with physical limitations persisting during the first 3 months and psychosocial recovery occurring progressively thereafter [19]. Because clinician-rated function correlates only weakly to moderately with patient-reported quality of life [20], assessment should also address functional disability, social participation, and emotional well-being.

### 4.1. Interpretation of Pharmacological Evidence

The strongest pharmacological evidence did not support routine corticosteroid treatment for immediate postoperative facial nerve palsy. Roh and Park found no difference between prednisolone and placebo, with 43 of 44 evaluable participants (98%) recovering completely by 6 months across the two groups combined [16]. This agrees with the review by Varadharajan et al., in which neither randomized trial showed a significant benefit from corticosteroids [3], and with the cohort reported by Tung et al. [18]. Evidence from Bell’s palsy should not be extrapolated directly because idiopathic inflammatory neuropathy within the facial canal differs from immediate postoperative dysfunction caused by traction, manipulation, ischemia, or injury to extratemporal branches. Delayed-onset palsy after initially preserved function may represent a distinct process. Proposed mechanisms include postoperative neural edema or compression, local inflammation, and reactivation of latent herpes-family viruses triggered by surgical stress [6,7]. The symptom-free interval and initially intact function make these mechanisms more plausible than direct intraoperative transection, although they remain unproven in the post-parotidectomy population. Management may therefore differ from that of immediate weakness: hematoma, infection, compressive collection, marked edema, and tumor-related complications should first be excluded; ocular protection should be instituted when eye closure is impaired; and corticosteroid or antiviral therapy should be regarded as empirical rather than established care. The two available case reports described complete or near-complete recovery after corticosteroids, with an antiviral added in one case [6,7], but they cannot define natural history or isolate either treatment effect. Future studies should analyze immediate and delayed presentations separately because their mechanisms, prognosis, and treatment response may differ.

### 4.2. Rehabilitation and Facial Exercise Therapy

Rehabilitation was the most frequently reported approach. Infante-Cossio et al. found no significant difference between supervised rehabilitation and structured mirror exercises performed at home [5]. The absence of a statistically significant difference does not establish equivalence. A well-taught home program may be a practical option, but the trial does not exclude a role for specialist rehabilitation for severe weakness, impaired motor control, ocular or oral dysfunction, synkinesis, poor adherence or incomplete recovery. Treatment should reflect baseline severity, functional impairment, recovery trajectory and patient goals. Wider evidence remains uncertain. A review of physical therapy for iatrogenic facial paralysis included 313 patients, 166 after parotidectomy, and generally reported improvement without clear harm; heterogeneity precluded comparisons between approaches [11]. Another review supported facial exercises in early and chronic palsy, but most evidence concerned Bell’s palsy [21]. Boschetti et al. associated early Kabat rehabilitation with faster short-term improvement, particularly in patients with House–Brackmann grade IV or V dysfunction [17]. Because treatment was not randomized, residual confounding and selection bias remain possible.

Molinari et al. reported progressive improvement during neuromuscular retraining, mainly within 6 months but continuing through 18 months in some patients [4]. The program combined selective symmetrical movements, massage, mirror feedback and daily practice. Without an untreated comparator, and with concurrent pharmacological treatment permitted, improvement cannot be attributed solely to retraining. Home exercises, supervised therapy, Kabat facilitation and neuromuscular retraining also differ in content, intensity and burden; future studies should describe each component rather than use broad labels such as “physiotherapy” or “facial exercises.”

### 4.3. Electrical Stimulation and Multimodal Interventions

Electrical stimulation and MRT were each reported in patients who improved during multimodal rehabilitation [9,10], so their independent effects could not be isolated. More broadly, evidence for electrical stimulation in peripheral facial paralysis varies by injury mechanism, treatment parameters and timing, and remains insufficient to establish it as a primary treatment [22]. Concerns include mistargeted reinnervation and synkinesis. A later review found no consistent increase in synkinesis with transcutaneous stimulation, but the available studies were few and heterogeneous [23]. In post-parotidectomy paresis, evidence was limited to the case reported by Alwash [10]; the absence of adverse events in one patient cannot establish safety across protocols or injury severities. Future studies should report electrode placement, waveform, frequency, pulse duration, intensity, session duration and treatment timing, with validated long-term assessment of synkinesis. MRT was likewise described in only one case and was combined with conventional physiotherapy [9]. No controlled or large-scale clinical evidence supporting MRT was identified in this review. Consequently, the observed improvement cannot be attributed to MRT, and its effectiveness and safety remain uncertain.

### 4.4. Outcome Measurement and Clinical Implications

The House–Brackmann (HB) system grades facial function from I (normal function) to VI (total paralysis), with grades II–V indicating progressively greater dysfunction [12].

All eight studies used the House–Brackmann scale, allowing a basic comparison of global function. However, the scale is relatively insensitive to regional change and does not fully capture synkinesis or patient-experienced disability. A review of facial grading instruments found strong reproducibility and greater regional detail for Sunnybrook, whereas House–Brackmann was simpler but less sensitive to subtle differences [24]. Future studies should therefore combine validated clinician-rated measures with patient-reported outcomes. The weak association between motor grade and social quality of life indicates that motor recovery does not ensure psychosocial recovery [20]. Instruments such as the Facial Disability Index or Facial Clinimetric Evaluation Scale can assess eating, drinking, communication, social confidence, and emotional well-being. Synkinesis should also be measured explicitly; international recommendations support standardized clinical and patient-reported assessment and facial biofeedback training when it develops [25].

### 4.5. Practical Management Considerations

Practical care begins with early documentation of facial function, exclusion of reversible postoperative complications, and protection of the ocular surface when eyelid closure is incomplete. Lubricating drops or ointment and temporary eyelid taping or moisture protection may be required, with prompt ophthalmologic assessment for ocular pain, redness, visual change, or suspected exposure keratopathy [26]. Home exercise programs should be taught with clear written or video instructions, mirror feedback, a realistic schedule, and follow-up to assess technique and adherence; overactivity or poorly targeted movement should be avoided. Supervised therapy can provide individualized feedback and progression, but repeated visits create direct costs and time or travel burdens for patients and services. The available trial was not designed as a cost-effectiveness or equivalence study, so the lower resource use of home exercises should not be interpreted as equal clinical efficacy. A pragmatic stepped approach may use a well-taught home program for uncomplicated paresis and specialist facial rehabilitation for severe, persistent, functionally disabling, or poorly controlled deficits. Although intraoperative facial nerve monitoring (IOFNM) concerns nerve identification, prevention, and perioperative risk assessment rather than treatment of established paresis, it provides relevant clinical context. A recent systematic review of eight observational studies (632 patients, including 193 with postoperative facial palsy) found that changes in amplitude or stimulation threshold were associated with dysfunction in some studies, but the predictive value of IOFNM parameters remained inconclusive; an amplitude reduction greater than 50% may identify patients at increased risk of postoperative dysfunction [27]. These findings support careful documentation of monitoring parameters without implying that IOFNM treats an established deficit. Branch-specific examination is also important because the marginal mandibular branch is the branch most frequently affected after parotidectomy [1]. Weakness may manifest as lower-lip asymmetry and impaired oral competence despite a relatively favorable global HB grade; regional documentation can therefore complement global grading and help target rehabilitation.

### 4.6. Future Research

Future studies should distinguish immediate from delayed dysfunction and document facial nerve preservation. Surgical extent, tumor pathology, revision surgery, baseline grade and intraoperative monitoring findings should be reported consistently. Adequately powered multicenter trials should compare clearly defined rehabilitation strategies, including structured home exercises versus specialist retraining, Kabat versus conventional therapy, and rehabilitation with versus without electrical stimulation. Treatment timing, frequency, intensity and duration also require evaluation. Future pharmacological trials should use adequate sample sizes and prespecified immediate, delayed or inflammatory presentations; the existing small trial does not exclude clinically relevant effects in specific subgroups. A core outcome set should include regional facial function, time to meaningful improvement and complete recovery, ocular and oral function, synkinesis and residual asymmetry, facial disability and quality of life, adherence and acceptability, and adverse events.

Follow-up should extend for at least 12 months and preferably 18–24 months in patients with incomplete recovery. Reporting outcomes at common postoperative intervals would facilitate comparison across studies and may permit future quantitative synthesis. Prospective multicenter registries could further define the natural history of postoperative facial nerve paresis and identify subgroups most likely to benefit from active rehabilitation.

### 4.7. Strengths and Limitations

This review focused specifically on treatment after facial nerve dysfunction, separating recovery strategies from prevention, surgical technique and risk prediction. PubMed, Embase and Scopus were searched from inception; two reviewers independently selected and appraised studies, and data charted by one reviewer were verified by a second. Including different designs enabled both established and emerging interventions to be mapped. Limitations include restriction to English-language journal articles and exclusion of gray literature, with no backward or forward citation tracking or trial-registry searches. Only eight studies were eligible, four were case reports, sample sizes were generally small and only three studies had comparators. Heterogeneous protocols, baseline severity, outcomes and assessment times precluded meta-analysis and limited direct comparisons. Most studies could not separate treatment effects from spontaneous recovery, and concurrent therapies were common. Adherence, adverse events and patient-reported outcomes were inconsistently documented. The findings should therefore be interpreted as a map of reported management strategies and recovery outcomes, not as evidence of comparative effectiveness. One potentially relevant report could not be retrieved, which may have affected the completeness of the evidence map. In two included case reports, nerve continuity was inferred rather than explicitly documented in the extracted operative information. These cases should not determine conclusions for patients with confirmed nerve preservation. The 211-patient total combines study-specific evaluable populations and is not a pooled estimate of treatment effect. Funding of the included studies was not systematically charted and its potential influence could not be assessed.

## 5. Conclusions

Postoperative facial nerve paresis following nerve-preserving parotidectomy generally has a favorable recovery trajectory, although the time required for recovery varies according to the initial severity of dysfunction and may extend beyond the first postoperative year. Reported management strategies include corticosteroids, antiviral therapy, structured home-based facial exercises, supervised rehabilitation, Kabat rehabilitation, neuromuscular retraining, proprioceptive neuromuscular facilitation, electrical stimulation and MRT.

The placebo-controlled trial did not demonstrate that prednisolone accelerated recovery or increased the probability of complete recovery in immediate postoperative facial nerve dysfunction. Similarly, supervised facial rehabilitation has not demonstrated superiority over a structured home-based mirror exercise program. Early Kabat rehabilitation and neuromuscular retraining may be associated with faster or continued functional improvement, particularly in patients with more severe deficits, but these findings derive from nonrandomized or uncontrolled studies and remain susceptible to selection bias, confounding and spontaneous recovery. Evidence supporting corticosteroid and antiviral treatment for delayed-onset palsy, electrical stimulation and MRT is limited to individual case reports. These approaches should therefore be regarded as exploratory rather than established standards of care. Clinical management considerations include early and standardized assessment of facial nerve function, exclusion of reversible postoperative complications, appropriate ocular and functional care, patient education and rehabilitation tailored to baseline severity, functional impairment and recovery trajectory. Structured home exercises may be appropriate for uncomplicated paresis, whereas specialist facial rehabilitation may be considered for severe, persistent or functionally disabling deficits. These clinical considerations are not a validated treatment pathway derived from this review, and no single therapeutic strategy has established superiority.

Future multicenter comparative studies should use standardized intervention protocols, validated clinician-rated and patient-reported outcomes, systematic safety assessment and sufficiently long follow-up. Such research is necessary to determine whether specific treatments improve recovery beyond the generally favorable natural course of postoperative facial nerve paresis when the facial nerve has been anatomically preserved.

## Figures and Tables

**Table 1:** **Characteristics of the included studies.** The table summarizes study location and design, sample characteristics, surgical context, baseline postoperative facial nerve dysfunction, outcome instruments and follow-up duration. Abbreviations: FDI, Facial Disability Index; HB, House–Brackmann; ICF, International Classification of Functioning, Disability and Health; MMT, manual muscle testing; SAQ, Synkinesis Assessment Questionnaire; Sunnybrook, Sunnybrook Facial Grading System [4,5,6,7,9,10,16,17].

Study	Country	Study Design	Sample Characteristics	Surgical Context and Baseline Facial Nerve Dysfunction	Outcome Instruments	Follow-Up
Roh and Park (2008) [16]	Republic of Korea	Randomized, double-blind, placebo-controlled trial	45 randomized; 44 evaluable (22 prednisolone, 22 placebo); mean age 51 vs. 50 years	Partial, superficial, or total parotidectomy; all facial nerve branches preserved; HB grade II–IV palsy, mainly involving one or two facial regions	HB grade	1, 3, and 6 months; one persistent case followed to 18 months
Infante-Cossio et al. (2016) [5]	Spain	Prospective randomized controlled trial	79 randomized; 61 with paresis at week 1; moderate-to-severe subgroup *n* = 45; mean age 48 years (range 24–81)	Conventional superficial parotidectomy with preservation of all facial nerve branches; dysfunction assessed at postoperative week 1	HB global and branch-specific scores	1, 3, 6, and 12 months
Boschetti et al. (2022) [17]	Italy	Retrospective case–control study	56 participants (28 Kabat rehabilitation, 28 controls); mean age 53.8 vs. 54.0 years	Extracapsular dissection or partial, superficial, or total parotidectomy; severe palsy (HB IV–V) at postoperative day 7	HB grade	Postoperative day 7 and 1, 3, and 6 months
Ostrowski et al. (2024) [6]	USA	Case report	One 70-year-old man	Total parotidectomy with the facial nerve preserved and electrically responsive; delayed HB VI paralysis on postoperative day 12	HB grade	10 weeks
Molinari et al. (2024) [4]	Italy	Retrospective multicenter cohort study	46 participants; mean age 54 years (range 17–86)	Nerve-sparing partial, subtotal, total, or revision parotid surgery; 39.1% had HB IV–VI palsy at discharge	HB and Sunnybrook scales	2, 6, 12, 18, and 24 months
Dharwadkar et al. (2025) [9]	India	Case report	One 38-year-old man	Superficial parotidectomy; facial nerve preservation not explicitly documented; continuity inferred from the clinical course; postoperative day-5 HB IV palsy	HB grade, facial MMT, strength-duration curve, and ICF profile	3 weeks
Kuba et al. (2025) [7]	Japan	Case report	One 50-year-old woman	Parotid lobectomy with preservation of the zygomatic and buccal branches and normal post-resection stimulation; delayed HB V palsy on day 16	HB grade and electroneurography	4 months after onset; recurrence surveillance > 1 year
Alwash (2026) [10]	Iraq	Case report	One 57-year-old man	Total parotidectomy; facial nerve preservation not explicitly documented; continuity inferred from the clinical course; HB IV palsy at 4-week physiotherapy assessment	HB grade, FDI, facial MMT, and SAQ	6-week intervention and 1-year follow-up

**Table 2:** **Therapeutic strategies and reported recovery outcomes.** The table summarizes treatment components, timing and duration, comparators, facial nerve recovery, safety outcomes and interpretation of the evidence. Abbreviations: FDI, Facial Disability Index; HB, House–Brackmann; MMT, manual muscle testing. “Not reported” indicates that the article did not provide an explicit safety assessment or statement [4,5,6,7,9,10,16,17].

Study	Therapeutic Strategy	Treatment Protocol, Timing, and Duration	Comparator	Reported Recovery Outcomes	Safety Outcomes	Interpretation of the Evidence
Roh and Park (2008) [16]	Oral prednisolone	50 mg/day for 5 days, 30 mg/day for 3 days, and 10 mg/day for 2 days; initiated on postoperative day 1 or 2 after recognition of palsy	Placebo for 10 days	43/44 (98%) recovered completely by 6 months and 37/44 (84%) by 3 months; recovery rates did not differ between groups	One minor steroid-related symptom; no major adverse effects	The only placebo-controlled pharmacological trial found no benefit of prednisolone in accelerating recovery
Infante-Cossio et al. (2016) [5]	Home mirror exercises versus supervised facial rehabilitation	Home group: 10 facial exercises in front of a mirror five times/day. Supervised group with moderate-to-severe paresis: ten weekly 45-min sessions of exercises and external/intraoral massage plus home exercises	Active comparator	No differences in prevalence, magnitude, duration, or recovery of paresis; complete facial mobility recovery occurred within 12 months in both groups	No treatment-related harms reported	No statistically significant between-group differences were detected; equivalence or non-inferiority was not established. Supervised therapy used more clinical resources.
Boschetti et al. (2022) [17]	Early Kabat rehabilitation	Initiated on postoperative day 7; three sessions/week for 12–24 weeks using diagonal and spiral proprioceptive neuromuscular facilitation movements of facial muscle groups	Non-Kabat standard management	At 3 months, HB grade was lower by 0.71 on average; probability of a high HB grade decreased by approximately 13%; 45% of participants with baseline HB V reached HB I versus none of the controls	Not reported	Early Kabat rehabilitation may accelerate short-term recovery, although the nonrandomized design limits causal inference
Ostrowski et al. (2024) [6]	High-dose oral prednisone	Prednisone 20 mg three times/day for 7 days, followed by a 3-day taper; a second course was administered after partial improvement; ocular lubrication was also prescribed	None	HB VI improved to HB II by 10 weeks	Not reported	Improvement followed treatment, but efficacy cannot be separated from spontaneous recovery in a single case
Molinari et al. (2024) [4]	Facial neuromuscular retraining	Selective small, slow, symmetrical movements, facial massage, and mirror feedback; taught by a speech therapist and practiced daily at home; initiated a mean of 15 days after surgery	No untreated control	Mean Sunnybrook score increased from 54 at baseline to 76.5 at 2 months and 95.4 at 6 months; complete recovery was reported in 72% of patients at 12 months, 85% at 18 months, and 89.1% at 24 months	No rehabilitation-related harms reported	Most improvement occurred within 6 months, with further gains through 18 months; the uncontrolled design and medical co-interventions limit attribution
Dharwadkar et al. (2025) [9]	MRT plus conventional physiotherapy	MRT for 45 min once weekly for 3 weeks, applied to the cervical region and facial muscles, plus facial exercises, mirror therapy, proprioceptive neuromuscular facilitation, and functional exercises three times/day	None	HB IV improved to HB II; MMT of the frontalis, orbicularis oculi, and orbicularis oris improved from 2 to 4, with better eye closure, smiling, and pouting	No adverse effects or complications reported; assessment method not described	Improvement reported with multimodal treatment, but the specific contribution of MRT cannot be isolated
Kuba et al. (2025) [7]	Corticosteroid-antiviral combination	Intravenous prednisolone sodium succinate 100 mg/day tapered to 40 mg/day over 1 week, followed by an oral taper; intravenous acyclovir 5 mg/kg every 8 h for 1 week	None	HB V palsy resolved completely 4 months after onset, with no recurrence during >1 year of surveillance	Not reported	Empirical combination treatment was followed by full recovery; the antiviral contribution remains uncertain
Alwash (2026) [10]	Proprioceptive neuromuscular facilitation combined with faradic electrical stimulation	Twelve sessions over 6 weeks: proprioceptive neuromuscular facilitation for approximately 20–30 min and faradic stimulation at 50 Hz for 20 min, twice weekly, plus daily 10-min home exercises	None	HB IV improved to HB II; FDI physical score increased from 44 to 93 and social/well-being score from 64 to 94; improvement persisted at 1 year, with residual platysma weakness	No adverse events, skin irritation, spasms, or treatment-induced synkinesis	Marked improvement was reported, but treatment effects cannot be separated from spontaneous recovery or individual intervention components

**Table 3:** **Critical appraisal of the included studies.** Methodological quality was evaluated independently by two reviewers using the Joanna Briggs Institute checklist appropriate to each study design. Studies were not excluded on the basis of appraisal findings. Abbreviations: JBI, Joanna Briggs Institute; N/A, not applicable; RCT, randomized controlled trial. Counts represent the final consensus ratings of the two reviewers. Checklist items were rated Yes, No, Unclear, or N/A; no aggregate quality threshold was used [4,5,6,7,9,10,16,17].

Study	Study Design	JBI Checklist	Yes	No	Unclear	N/A	Interpretive Summary
Roh and Park (2008) [16]	Randomized, double-blind, placebo-controlled trial	JBI RCT	9	2	2	0	Generally strong outcome assessment and blinding; random-sequence generation and allocation concealment were insufficiently described, and one randomized participant was excluded from analysis.
Infante-Cossio et al. (2016) [5]	Prospective randomized controlled trial	JBI RCT	10	2	0	1	Adequate randomization, concealment, blinded outcome assessment, and intention-to-treat analysis; participants and therapists could not be blinded.
Boschetti et al. (2022) [17]	Retrospective case–control study	JBI Case–Control	9	1	0	0	Standardized assessment and adjusted analyses were reported, but treatment allocation was self-selected and groups were not individually matched, leaving residual confounding.
Molinari et al. (2024) [4]	Retrospective multicenter single-cohort study	JBI Cohort	7	1	1	2	Validated repeated outcome measures and multivariable analyses were used; absence of an untreated comparator and incompletely described attrition limit causal attribution.
Ostrowski et al. (2024) [6]	Case report	JBI Case Report	7	1	0	0	Clinical course and intervention were described clearly, but treatment-related adverse events were not explicitly reported and spontaneous recovery cannot be excluded.
Dharwadkar et al. (2025) [9]	Case report	JBI Case Report	8	0	0	0	Detailed intervention and multidomain outcomes; no side effects or complications were reported. The single-case design limits safety conclusions, and the contribution of MRT cannot be isolated.
Kuba et al. (2025) [7]	Case report	JBI Case Report	7	1	0	0	Detailed diagnostic work-up, treatment, and follow-up; adverse-event reporting was absent and causality cannot be inferred.
Alwash (2026) [10]	Case report	JBI Case Report	8	0	0	0	Complete clinical timeline, intervention, follow-up, and safety reporting; the single-case design and spontaneous recovery remain major limitations.

## Data Availability

All data extracted and analyzed in this scoping review are reported in the article and its Supplementary Materials. No new primary data were generated.

## Supplementary Materials

The following supporting information can be downloaded at: https://www.mdpi.com/article/10.3390/jcm15218417/s1. The supplementary file includes the PRISMA-ScR checklist; Supplementary Material S1, complete electronic search strategies; Supplementary Material S2, eligibility criteria; Supplementary Material S3, reports excluded after full-text assessment; Supplementary Material S4, data-charting framework; Supplementary Material S5, design-specific JBI critical appraisal results; Table S1, detailed characteristics of the included studies; and Table S2, therapeutic protocols and recovery outcomes. References [4,5,6,7,9,10,14,15,16,17,28,29,30,31] are cited in the supplementary file.

## Abbreviations

FDI, Facial Disability Index; FN, facial nerve; FNP, facial nerve palsy; HB, House–Brackmann; ICF, International Classification of Functioning, Disability and Health; IOFNM, intraoperative facial nerve monitoring; IV, intravenous; MRT, Matrix Rhythm Therapy; MMT, manual muscle testing; PNF, proprioceptive neuromuscular facilitation; PRISMA-ScR, Preferred Reporting Items for Systematic Reviews and Meta-Analyses extension for Scoping Reviews; SAQ, Synkinesis Assessment Questionnaire; SB, Sunnybrook Facial Grading System.
