# Supplementary material for: Management of Facial Nerve Paresis After Nerve-Preserving Parotidectomy: A Review
Source: J Clin Med. 2026 Oct 29;15(21):8417. doi: 10.3390/jcm15218417 (PMC13764521; doi:10.3390/jcm15218417)
Supplement: Supplementary file 1 [file jcm-15-08417-s001.zip › jcm-4656216-supplementary.pdf]

Supplementary Material

Management of Facial Nerve Paresis After Nerve-Preserving Parotidectomy: A Review

This file contains the PRISMA-ScR checklist, Supplementary Materials S1–S5, and Tables S1–S2. Reference numbers correspond to the main manuscript. The review maps eight studies and does not establish comparative treatment superiority.

PRISMA ScR checklist

Adapted from Tricco et al. [15]. Locations refer to the accompanying proofread manuscript; section and table labels remain the primary locators if pagination changes.

Section / topic	Item	Checklist requirement	Location / reporting status
Title	1	Identify the report as a scoping review.	p. 1: title says Review, not scoping review; method identified in the abstract and Section 2.1 (p. 3). Item not fully met in the title.
Structured summary	2	Provide a structured summary that includes (as applicable): background, objectives, eligibility criteria, sources of evidence, charting methods, results, and conclusions that relate to the review questions and objectives.	p. 1, Abstract
Rationale	3	Describe the rationale for the review in the context of what is already known. Explain why the review questions/objectives lend themselves to a scoping review approach.	p. 2, Introduction
Objectives	4	Provide an explicit statement of the questions and objectives being addressed with reference to their key elements (e.g., population or participants, concepts, and context) or other relevant key elements used to conceptualize the review questions and/or objectives.	p. 3, Sections 1.1–1.2
Protocol and registration	5	Indicate whether a review protocol exists; state if and where it can be accessed (e.g., a Web address); and if available, provide registration information, including the registration number.	p. 3, Section 2.1
Eligibility criteria	6	Specify characteristics of the sources of evidence used as eligibility criteria (e.g., years considered, language, and publication status), and provide a rationale.	pp. 3–4, Section 2.1.1; Supplementary Material S2
Information sources*	7	Describe all information sources in the search (e.g., databases with dates of coverage and contact with authors to identify additional sources), as well as the date the most recent search was executed.	p. 4, Sections 2.1.2 and 2.1.4
Search	8	Present the full electronic search strategy for at least 1 database, including any limits used, such that it could be repeated.	p. 4, Section 2.1.2; Supplementary Material S1
Selection of sources of evidence	9	State the process for selecting sources of evidence (i.e., screening and eligibility) included in the scoping review.	p. 4, Section 2.1.3
Data charting process	10	Describe the methods of charting data from the included sources of evidence (e.g., calibrated forms or forms that have been tested by the team before their use, and whether data charting was done independently or in duplicate) and any processes for obtaining and confirming data from investigators.	p. 4, Section 2.1.4; Supplementary Material S4
Data items	11	List and define all variables for which data were sought and any assumptions and simplifications made.	p. 5, Section 2.1.5; Supplementary Material S4

Section / topic	Item	Checklist requirement	Location / reporting status
Critical appraisal of individual sources of evidence	12	If done, provide a rationale for conducting a critical appraisal of included sources of evidence; describe the methods used and how this information was used in any data synthesis (if appropriate).	p. 5, Section 2.1.7
Synthesis of results	13	Describe the methods of handling and summarizing the data that were charted.	p. 5, Section 2.1.6
Selection of sources of evidence	14	Give numbers of sources of evidence screened, assessed for eligibility, and included in the review, with reasons for exclusions at each stage, ideally using a flow diagram.	p. 6, Section 3.1 and Figure 1; Supplementary Material S3
Characteristics of sources of evidence	15	For each source of evidence, present characteristics for which data were charted and provide the citations.	pp. 7–8, Section 3.2 and Table 1; Table S1
Critical appraisal within sources of evidence	16	If done, present data on critical appraisal of included sources of evidence (see item 12).	pp. 13–15, Section 3.4 and Table 3; Supplementary Material S5
Results of individual sources of evidence	17	For each included source of evidence, present the relevant data that were charted that relate to the review questions and objectives.	pp. 8–13, Section 3.3 and Table 2; Table S2
Synthesis of results	18	Summarize and/or present the charting results as they relate to the review questions and objectives.	pp. 15–16, Sections 3.5–3.7
Summary of evidence	19	Summarize the main results (including an overview of concepts, themes, and types of evidence available), link to the review questions and objectives, and consider the relevance to key groups.	pp. 16–19, Discussion
Limitations	20	Discuss the limitations of the scoping review process.	p. 19, Section 4.7
Conclusions	21	Provide a general interpretation of the results with respect to the review questions and objectives, as well as potential implications and/or next steps.	pp. 19–20, Conclusions
Funding	22	Describe sources of funding for the included sources of evidence, as well as sources of funding for the scoping review. Describe the role of the funders of the scoping review.	p. 20, Funding; p. 19, Section 4.7 (funding of included studies not systematically charted). Partially reported.

Terminology: information sources are the databases or other resources searched; sources of evidence are the reports or other evidence assessed. Data charting refers to data extraction in a scoping review. Critical appraisal refers to examining the validity, results, and relevance of evidence [14,15].

Supplementary Material S1. Complete electronic search strategies

Management of Facial Nerve Paresis After Nerve-Preserving Parotidectomy: A Review

PubMed

Database: MEDLINE

Platform: PubMed

Search date: 24 August 2026

Language: English

Date restriction: None

Records retrieved: 1157

Search version: PubMed v2

Full search strategy:

```
(
"Parotidectomy"[Mesh]
OR "Parotid Gland/surgery"[Mesh]
OR "Parotid Neoplasms/surgery"[Mesh]
OR parotidectom*[Title/Abstract]
OR "parotid surgery"[Title/Abstract]
OR "parotid gland surgery"[Title/Abstract]
OR "parotid resection"[Title/Abstract]
)
```

AND

```
(
"Facial Paralysis"[Mesh]
OR "Facial Nerve Injuries"[Mesh]
OR "facial nerve paresis"[Title/Abstract]
OR "facial nerve palsy"[Title/Abstract]
OR "facial nerve paralysis"[Title/Abstract]
OR "facial nerve weakness"[Title/Abstract]
OR "facial nerve dysfunction"[Title/Abstract]
OR "facial nerve injury"[Title/Abstract]
OR "facial nerve deficit"[Title/Abstract]
OR "facial nerve recovery"[Title/Abstract]
OR "facial nerve outcome"[Title/Abstract]
OR "facial nerve outcomes"[Title/Abstract]
OR "facial paresis"[Title/Abstract]
OR "facial palsy"[Title/Abstract]
OR "facial paralysis"[Title/Abstract]
OR "facial weakness"[Title/Abstract]
OR "facial dysfunction"[Title/Abstract]
)
AND english[Language]
```

Scopus

Database: Scopus

Platform: Elsevier

Search date: 24 August 2026

Language eligibility: English full texts selected during screening; no language-limit clause is present in the saved Scopus query.

Date restriction: None

Records retrieved: 1756

Full search strategy:

```
TITLE-ABS-KEY(
parotidectom*
OR "parotid surgery"
OR "parotid gland surgery"
OR "parotid resection"
)
```

AND

```
TITLE-ABS-KEY(
"facial nerve paresis"
OR "facial nerve palsy"
OR "facial nerve paralysis"
OR "facial nerve weakness"
OR "facial nerve dysfunction"
OR "facial nerve injury"
OR "facial nerve injuries"
OR "facial nerve deficit"
OR "facial nerve recovery"
OR "facial nerve outcome"
OR "facial nerve outcomes"
OR "facial paresis"
OR "facial palsy"
OR "facial paralysis"
OR "facial weakness"
OR "facial dysfunction"
)
```

Embase

Database: Embase

Platform: Embase.com, Elsevier

Search date: 24 August 2026

Language: English

Date restriction: None

Records retrieved: 1512

Full search strategy:

```
(
'parotidectomy'/exp
OR parotidectom*:ti,ab,kw
OR 'parotid surgery':ti,ab,kw
OR 'parotid gland surgery':ti,ab,kw
OR 'parotid resection':ti,ab,kw
)
```

AND

```
(
'facial paralysis'/exp
OR 'facial nerve injury'/exp
OR 'facial nerve paresis':ti,ab,kw
OR 'facial nerve palsy':ti,ab,kw
OR 'facial nerve paralysis':ti,ab,kw
OR 'facial nerve weakness':ti,ab,kw
OR 'facial nerve dysfunction':ti,ab,kw
OR 'facial nerve injury':ti,ab,kw
OR 'facial nerve injuries':ti,ab,kw
OR 'facial nerve deficit':ti,ab,kw
OR 'facial nerve recovery':ti,ab,kw
OR 'facial nerve outcome':ti,ab,kw
OR 'facial nerve outcomes':ti,ab,kw
OR 'facial paresis':ti,ab,kw
OR 'facial palsy':ti,ab,kw
OR 'facial paralysis':ti,ab,kw
OR 'facial weakness':ti,ab,kw
OR 'facial dysfunction':ti,ab,kw
)
AND [english]/lim
```

Supplementary Material S2. Eligibility criteria structured according to the Population-Concept-Context framework

Therapeutic management of postoperative facial nerve paresis following nerve-preserving parotidectomy

Review question: What therapeutic strategies have been reported for the management of postoperative facial nerve paresis following nerve-preserving parotidectomy, and what recovery outcomes have been documented?

PCC element	Inclusion definition	Key exclusions
Population	Patients of any age with postoperative facial nerve paresis or paralysis after parotidectomy in which the facial nerve was anatomically preserved.	Exclude nerve sacrifice, transection, grafting, or reconstruction; exclude pre-existing palsy unless postoperative treatment data are separable.
Concept	Therapeutic management of established postoperative facial nerve dysfunction, including pharmacological treatment, exercise-based or neuromuscular rehabilitation, physical modalities, and multimodal interventions; together with recovery, functional, patient-reported, residual-deficit, and safety outcomes.	Exclude studies limited to incidence, risk prediction, surgical technique, monitoring, or prevention without management of an established postoperative deficit.
Context	Any inpatient, outpatient, rehabilitation, or home-based setting after nerve-preserving partial, superficial, subtotal, total, or revision parotidectomy for benign or malignant parotid disease.	Exclude non-parotid causes when data are not separable.

Additional source-level eligibility criteria

Original full-text journal articles published in English.

Randomized and nonrandomized trials, cohort studies, case-control studies, case series, and case reports.

At least one post-treatment facial nerve recovery, functional, patient-reported, residual-deficit, time-to-recovery, or safety outcome.

Mixed populations only when relevant post-parotidectomy data could be identified separately.

Excluded source types

Reviews, guidelines, editorials, letters without original clinical data, conference abstracts, theses, dissertations, and gray literature.

Non-English reports and reports for which the full text could not be retrieved.

Operational rule: Information was not inferred when it could not be established reliably. Facial nerve preservation could be accepted as clinically implicit only when the operative and recovery course supported nerve continuity and no sacrifice or reconstruction was reported.

Supplementary Material S3. Reports excluded after full-text assessment

Two reports were excluded after assessment of the complete article; reasons are stated at the report level.

Report	Full citation title / DOI	Primary reason	Detailed rationale
Banquer de Borello (1973) [28]	Treatment of peripheral facial paralysis and post-parotidectomy with ACTH-GEL. Report of 17 cases. Revista de la Asociación Odontológica Argentina. 1973;61(1):5–6. PMID: 4352044.	Non-English publication	The full text was successfully retrieved but was published entirely in Spanish. Because the eligibility criteria were restricted to full-text journal articles published in English, the report was excluded after full-text assessment.
Lee et al. (2002) [29]	The efficacy of corticosteroids in postparotidectomy facial nerve paresis. DOI: 10.1097/00005537-200211000-00009	Preventive/perioperati ve intervention	Dexamethasone was administered immediately before surgery and during the first 16 postoperative hours, before postoperative paresis was established. The intervention was therefore perioperative prophylaxis rather than treatment initiated for established postoperative facial nerve paresis.

Retrieval note: Targino da Costa et al. (2024) [30], "Post-parotidectomy facial nerve rehabilitation outcomes: Comparison between benign and malignant neoplasms," was sought but could not be retrieved. It was therefore not assessed for eligibility and is not counted among the two reports excluded after full-text assessment.

Supplementary Material S4. Data-charting framework and definitions of extracted variables

The form was piloted, refined iteratively, and checked by both reviewers against the full text.

Domain	Variable	Operational definition / charting rule
Bibliographic	Study ID / citation	First author, publication year, title, journal, DOI, and country.
Methods	Study design	Design as reported and verified from methods: RCT, cohort, case-control, case series, or case report.
Methods	Clinical setting	Institutional and care context, when reported.
Population	Analysis sample	Number with postoperative paresis contributing to therapeutic outcomes; total randomized/enrolled also retained when relevant.
Population	Age and sex	Summary or individual demographic characteristics as reported; discrepancies retained according to the detailed article text.
Clinical	Indication / pathology	Parotid disease and histological diagnosis.
Surgery	Parotidectomy	Type and extent: partial, superficial, subtotal, total, revision, or other specified operation.
Surgery	Facial nerve preservation	Explicit operative preservation or clinically supported continuity with no reported sacrifice/reconstruction.
Baseline deficit	Onset and timing	Immediate or delayed onset and postoperative time at first documented paresis.
Baseline deficit	Severity and distribution	Global grade, branch pattern, and affected facial regions as originally reported.
Intervention	Strategy category	Pharmacological; exercise-based/neuromuscular rehabilitation; physical modality; multimodal.
Intervention	Components	Named treatment elements, co-interventions, home program, and ocular care.
Intervention	Dose / intensity	Drug dose and taper; session frequency and duration; device parameters; exercise repetitions.
Intervention	Timing / duration	Time from surgery or paresis onset to initiation and total treatment period.
Comparison	Comparator	Placebo, active comparator, standard management, untreated control, or none.
Outcomes	Facial nerve instruments	HB, Sunnybrook, FDI, MMT, SAQ, electroneurography, strength-duration curve, ICF, or other reported measure.
Outcomes	Follow-up	Assessment time points and maximum reported follow-up.
Outcomes	Recovery	Direction and magnitude of change, complete/incomplete recovery, proportion recovered, and time to recovery.

Domain	Variable	Operational definition / charting rule
Outcomes	Residual outcomes	Persistent weakness, branch-specific deficit, synkinesis, functional limitation, or recurrence of palsy.
Outcomes	Patient-reported outcomes	Physical and social function, well-being, quality of life, or other patient-reported measure.
Safety	Adverse events / tolerability	Treatment-related harms, irritation, spasms, synkinesis, complications, or explicit absence; otherwise NR.
Interpretation	Authors' conclusions	Principal interpretation stated by the study authors.
Interpretation	Review-level limitation	Design-specific uncertainty, spontaneous recovery, confounding, co-intervention, selection, attrition, or imprecision.
Data handling	NR	Not reported in the article; missing information was not inferred.
Data handling	Scale handling	Scores retained as originally reported; no conversion to a common facial grading scale.

Supplementary Material S5. Design-specific JBI critical appraisal results

The design-specific JBI tools [31] are reproduced here as the item sets used in the appraisal. Study citations use the reference numbering of the main manuscript.

Two reviewers independently applied the appropriate JBI checklist and resolved discrepancies by consensus. Appraisal did not determine eligibility.

JBI checklist for randomized controlled trials

Study	Item	JBI criterion	Final consensus	Rationale	Source location
Roh and Park (2008) [16]	1	Was true randomization used for assignment of participants to treatment groups?	Unclear	The article states that patients were assigned randomly but does not describe sequence generation.	Methods—Patients and study design
Roh and Park (2008) [16]	2	Was allocation to treatment groups concealed?	Unclear	Allocation concealment is not described.	Methods—Patients and study design
Roh and Park (2008) [16]	3	Were treatment groups similar at baseline?	Yes	Age, sex, baseline HB severity and affected areas were comparable.	Table 2
Roh and Park (2008) [16]	4	Were participants blind to treatment assignment?	Yes	The trial is described as double-blind and used similarly formulated placebo.	Methods—study design and intervention
Roh and Park (2008) [16]	5	Were those delivering treatment blind to treatment assignment?	Yes	Double-blind placebo-controlled design supports blinding of treatment delivery, although operational details are limited.	Methods—study design
Roh and Park (2008) [16]	6	Were outcomes assessors blind to treatment assignment?	Yes	Two experts independently graded outcomes while unaware of group assignment and assessment stage.	Methods—Outcome measurements
Roh and Park (2008) [16]	7	Were treatment groups treated identically other than the intervention of interest?	Yes	Both groups received identically scheduled 10-day regimens, differing only in prednisolone versus lactose placebo.	Methods—intervention
Roh and Park (2008) [16]	8	Was follow-up complete and, if not, were differences adequately described and analyzed?	No	One of 23 prednisolone participants was lost and excluded; no missing-data sensitivity analysis was reported.	Results; Figure 1
Roh and Park (2008) [16]	9	Were participants analyzed in the groups to which they were randomized?	No	The participant lost after randomization was not included in the final analysis.	Results
Roh and Park (2008) [16]	10	Were outcomes measured in the same way for treatment groups?	Yes	HB grading and visit schedule were the same in both groups.	Methods—Outcome measurements
Roh and Park (2008) [16]	11	Were outcomes measured in a reliable way?	Yes	Validated HB grading was independently performed by two blinded experts with reassessment of larger discrepancies.	Methods—Outcome measurements

Study	Item	JBI criterion	Final consensus	Rationale	Source location
Roh and Park (2008) [16]	12	Was appropriate statistical analysis used?	Yes	Between-group categorical and continuous comparisons were appropriate for the reported data.	Methods—Statistical analysis
Roh and Park (2008) [16]	13	Was the trial design appropriate and any deviations accounted for?	Yes	Individual randomized parallel placebo-controlled design was appropriate.	Methods
Infante-Cossio et al. (2016) [5]	1	Was true randomization used for assignment of participants to treatment groups?	Yes	A computer-generated random-number table was prepared by an independent statistician.	Methods—randomization
Infante-Cossio et al. (2016) [5]	2	Was allocation to treatment groups concealed?	Yes	Assignments were placed in sealed, masked, numbered envelopes opened after postoperative eligibility confirmation.	Methods—randomization
Infante-Cossio et al. (2016) [5]	3	Were treatment groups similar at baseline?	Yes	Groups were reported as homogeneous at baseline.	Results—participant characteristics
Infante-Cossio et al. (2016) [5]	4	Were participants blind to treatment assignment?	No	The authors explicitly state that patients were not blinded.	Discussion—limitations
Infante-Cossio et al. (2016) [5]	5	Were those delivering treatment blind to treatment assignment?	No	Surgeons and therapists were not blinded.	Discussion—limitations
Infante-Cossio et al. (2016) [5]	6	Were outcomes assessors blind to treatment assignment?	Yes	The same independent observer, blinded to allocation, performed follow-up assessments.	Methods—follow-up assessment
Infante-Cossio et al. (2016) [5]	7	Were treatment groups treated identically other than the intervention of interest?	Yes	The surgical procedure and co-management were standardized; intervention-related contact differed as required by design.	Methods—surgery and interventions
Infante-Cossio et al. (2016) [5]	8	Was follow-up complete and, if not, were differences adequately described and analyzed?	Yes	Participant flow was described and the primary analysis included all 79 randomized participants.	Figure 1; Statistical analysis
Infante-Cossio et al. (2016) [5]	9	Were participants analyzed in the groups to which they were randomized?	Yes	An intention-to-treat analysis included all randomized participants.	Methods—Statistical analysis
Infante-Cossio et al. (2016) [5]	10	Were outcomes measured in the same way for treatment groups?	Yes	The same HB-based assessment and time points were used for both groups.	Methods—outcome assessment
Infante-Cossio et al. (2016) [5]	11	Were outcomes measured in a reliable way?	Yes	A standardized facial grading system was applied by the same blinded assessor.	Methods—outcome assessment
Infante-Cossio et al. (2016) [5]	12	Was appropriate statistical analysis used?	Yes	Time-to-recovery and group comparisons were analyzed using appropriate survival and non-parametric methods.	Methods—Statistical analysis

Study	Item	JBI criterion	Final consensus	Rationale	Source location
Infante-Cossio et al. (2016) [5]	13	Was the trial design appropriate and any deviations accounted for?	N/A	No material deviation from a standard individual parallel-group randomized design required adjustment.	Methods

JBI checklist for case-control studies

Study	Item	JBI criterion	Final consensus	Rationale	Source location
Boschetti et al. (2022) [17]	1	Were the groups comparable other than the exposure/intervention of interest?	Yes	Groups were reported as homogeneous for sex, age, baseline HB grade and extent of surgery.	Methods; Table 2
Boschetti et al. (2022) [17]	2	Were cases and controls matched appropriately?	No	Participants were not individually matched; group membership reflected whether Kabat therapy was undertaken.	Methods; Discussion
Boschetti et al. (2022) [17]	3	Were the same criteria used for identification of cases and controls?	Yes	The same prespecified eligibility criteria and baseline HB IV–V requirement applied.	Methods—inclusion/exclusion criteria
Boschetti et al. (2022) [17]	4	Was exposure measured in a standard, valid and reliable way?	Yes	Kabat exposure was defined by an explicit protocol starting on postoperative day 7.	Methods—rehabilitation protocol
Boschetti et al. (2022) [17]	5	Was exposure measured in the same way for cases and controls?	Yes	Therapy status was identified from the same clinical records for both groups.	Methods
Boschetti et al. (2022) [17]	6	Were confounding factors identified?	Yes	Age, sex, baseline severity, pathology, tumor subsite and surgical extent were considered.	Methods; Tables 1–2
Boschetti et al. (2022) [17]	7	Were strategies to deal with confounding factors stated?	Yes	Multiple regression, difference-in-differences and logistic regression were used to address measured covariates.	Methods—Statistical analysis
Boschetti et al. (2022) [17]	8	Were outcomes assessed in a standard, valid and reliable way for cases and controls?	Yes	A single physician used HB grading at the same postoperative time points.	Methods—facial nerve assessment
Boschetti et al. (2022) [17]	9	Was the exposure period of interest long enough to be meaningful?	Yes	Follow-up through 6 months was clinically meaningful for early postoperative recovery.	Methods; Table 2
Boschetti et al. (2022) [17]	10	Was appropriate statistical analysis used?	Yes	The reported regression and difference-in-differences analyses were broadly appropriate, although residual confounding remains.	Methods—Statistical analysis

JBI checklist for cohort studies

Study	Item	JBI criterion	Final consensus	Rationale	Source location
Molinari et al. (2024) [4]	1	Were the two groups similar and recruited from the same population?	N/A	The study was a single treated cohort without an untreated comparison group.	Methods—Patients
Molinari et al. (2024) [4]	2	Were exposures measured similarly to assign people to exposed and unexposed groups?	N/A	All included participants received neuromuscular retraining.	Methods—rehabilitation program
Molinari et al. (2024) [4]	3	Was the exposure measured in a valid and reliable way?	Yes	The NMR program and initiation were explicitly described and delivered by a dedicated team.	Methods—rehabilitation program
Molinari et al. (2024) [4]	4	Were confounding factors identified?	Yes	Age, sex, severity, surgery type, nerve stimulation and medical co-treatment were examined.	Methods—Statistical analysis
Molinari et al. (2024) [4]	5	Were strategies to deal with confounding factors stated?	Yes	Repeated-measures and multivariable regression analyses assessed measured prognostic variables.	Methods—Statistical analysis
Molinari et al. (2024) [4]	6	Were participants free of the outcome at the start of the study?	Yes	All participants had postoperative palsy and had not achieved the recovery outcomes at rehabilitation baseline.	Eligibility; baseline assessment
Molinari et al. (2024) [4]	7	Were outcomes measured in a valid and reliable way?	Yes	HB and Sunnybrook scales were repeatedly applied by a specialist multidisciplinary team.	Methods—assessment
Molinari et al. (2024) [4]	8	Was follow-up time reported and sufficient to be long enough for outcomes to occur?	Yes	Follow-up was planned through 24 months, sufficient to capture delayed recovery.	Methods—follow-up
Molinari et al. (2024) [4]	9	Was follow-up complete and, if not, were reasons for loss described and explored?	Unclear	Follow-up availability varied across time points and reasons for incomplete observations were not fully detailed.	Results; study limitations
Molinari et al. (2024) [4]	10	Were strategies to address incomplete follow-up utilized?	No	No imputation, inverse weighting or sensitivity analysis for incomplete longitudinal follow-up was reported.	Statistical analysis
Molinari et al. (2024) [4]	11	Was appropriate statistical analysis used?	Yes	Repeated-measures testing and regression were broadly appropriate for the reported longitudinal associations.	Methods—Statistical analysis

JBI checklist for case reports

Study	Item	JBI criterion	Final consensus	Rationale	Source location
Ostrowski et al. (2024) [6]	1	Were the patient's demographic characteristics clearly described?	Yes	Age, sex, diagnosis and relevant clinical history were reported.	Case presentation
Ostrowski et al. (2024) [6]	2	Was the patient's history clearly described and presented as a timeline?	Yes	Surgery, normal early examinations, day-12 onset, treatment courses and follow-up were chronologically described.	Case presentation
Ostrowski et al. (2024) [6]	3	Was the current clinical condition on presentation clearly described?	Yes	Acute complete hemifacial paralysis was graded HB VI and accompanying findings were described.	Case presentation
Ostrowski et al. (2024) [6]	4	Were diagnostic tests or assessment methods and results clearly described?	Yes	Preoperative imaging, pathology, operative nerve preservation and clinical differential assessment were reported.	Case presentation
Ostrowski et al. (2024) [6]	5	Was the intervention or treatment procedure clearly described?	Yes	Prednisone dose, duration, taper, repeat course and ocular lubrication were reported.	Case presentation
Ostrowski et al. (2024) [6]	6	Was the post-intervention clinical condition clearly described?	Yes	Improvement to HB II at 10 weeks was documented.	Case presentation
Ostrowski et al. (2024) [6]	7	Were adverse events or unanticipated events identified and described?	No	Treatment-related adverse events were not explicitly assessed or reported.	Entire report
Ostrowski et al. (2024) [6]	8	Does the case report provide takeaway lessons?	Yes	The report emphasizes delayed palsy recognition, differential diagnosis and the uncertainty of treatment attribution.	Discussion/Conclusions
Dharwadkar et al. (2025) [9]	1	Were the patient's demographic characteristics clearly described?	Yes	Age, sex, tumor diagnosis and relevant history were described.	Case report
Dharwadkar et al. (2025) [9]	2	Was the patient's history clearly described and presented as a timeline?	Yes	Diagnostic work-up, surgery, postoperative palsy and three-week treatment course were presented chronologically.	Case report
Dharwadkar et al. (2025) [9]	3	Was the current clinical condition on presentation clearly described?	Yes	Facial asymmetry, movement deficits and HB IV status were described.	Clinical examination
Dharwadkar et al. (2025) [9]	4	Were diagnostic tests or assessment methods and results clearly described?	Yes	FNAC, HB, facial MMT, strength-duration testing and ICF assessment were reported.	Assessment; Tables 1–2
Dharwadkar et al. (2025) [9]	5	Was the intervention or treatment procedure clearly described?	Yes	MaRhyThe duration/frequency and concurrent conventional physiotherapy were described.	Intervention; Table 1

Study	Item	JBI criterion	Final consensus	Rationale	Source location
Dharwadkar et al. (2025) [9]	6	Was the post-intervention clinical condition clearly described?	Yes	Post-treatment HB, MMT, functional movements and ICF changes were described.	Follow-up; Table 2
Dharwadkar et al. (2025) [9]	7	Were adverse events or unanticipated events identified and described?	Yes	The Conclusion explicitly reports no side effects or complications after treatment. The method of adverse-event assessment is not described; this statement alone does not establish safety.	Conclusion
Dharwadkar et al. (2025) [9]	8	Does the case report provide takeaway lessons?	Yes	The authors identify the modality as preliminary and call for randomized trials.	Discussion/Conclusion
Kuba et al. (2025) [7]	1	Were the patient's demographic characteristics clearly described?	Yes	Age, sex, medical history and tumor presentation were described.	Case presentation
Kuba et al. (2025) [7]	2	Was the patient's history clearly described and presented as a timeline?	Yes	Surgery, early HB II deficit, pain, delayed HB V onset, treatment and four-month recovery were presented as a timeline.	Case presentation
Kuba et al. (2025) [7]	3	Was the current clinical condition on presentation clearly described?	Yes	Distribution, severity, pain and absence of infection, edema, hematoma or recurrence were detailed.	Case presentation
Kuba et al. (2025) [7]	4	Were diagnostic tests or assessment methods and results clearly described?	Yes	Imaging, pathology, nerve stimulation, serology, electroneurography and clinical examination were reported.	Case presentation
Kuba et al. (2025) [7]	5	Was the intervention or treatment procedure clearly described?	Yes	Intravenous steroid and acyclovir regimens followed by oral taper were described.	Treatment
Kuba et al. (2025) [7]	6	Was the post-intervention clinical condition clearly described?	Yes	Complete recovery at four months and surveillance beyond one year were reported.	Outcome/follow-up
Kuba et al. (2025) [7]	7	Were adverse events or unanticipated events identified and described?	No	Treatment-related adverse effects were not explicitly assessed or reported.	Entire report
Kuba et al. (2025) [7]	8	Does the case report provide takeaway lessons?	Yes	The report stresses investigation of alternative causes before empirical treatment.	Discussion/Conclusions
Alwash (2026) [10]	1	Were the patient's demographic characteristics clearly described?	Yes	Age, sex, diagnosis, surgery and relevant health history were reported.	Case report
Alwash (2026) [10]	2	Was the patient's history clearly described and presented as a timeline?	Yes	A figure and text present diagnosis, surgery, referral, treatment and one-year follow-up chronologically.	Figure 1; Case report
Alwash (2026) [10]	3	Was the current clinical condition on presentation clearly described?	Yes	Branch-specific weakness, HB IV, MMT and baseline FDI were described.	Physical assessment

Study	Item	JBI criterion	Final consensus	Rationale	Source location
Alwash (2026) [10]	4	Were diagnostic tests or assessment methods and results clearly described?	Yes	HB, FDI, MMT and synkinesis assessment were clearly reported, with the absence of electrodiagnostic testing acknowledged.	Assessment
Alwash (2026) [10]	5	Was the intervention or treatment procedure clearly described?	Yes	PNF, faradic stimulation parameters, session frequency/duration and home exercises were detailed.	Treatment
Alwash (2026) [10]	6	Was the post-intervention clinical condition clearly described?	Yes	Six-week and one-year functional, HB, FDI and residual-deficit outcomes were reported.	Results; Table 2
Alwash (2026) [10]	7	Were adverse events or unanticipated events identified and described?	Yes	The authors explicitly reported no skin irritation, spasms, adverse events or treatment-induced synkinesis.	Adverse events and safety
Alwash (2026) [10]	8	Does the case report provide takeaway lessons?	Yes	The report distinguishes observed improvement from causal efficacy and recommends controlled trials.	Limitations/Conclusion

Ratings: Yes = criterion met; No = criterion not met; Unclear = insufficient information; N/A = not applicable. No aggregate quality threshold was used.

Table S1. Detailed characteristics of the included studies

Detailed study-level information supporting the descriptive synthesis.

Study	Country	Design	Sample with paresis	Age	Pathology	Surgery / nerve preservation	Baseline postoperative deficit	Outcome instruments	Follow-up	DOI
Roh & Park (2008) [16]	Republic of Korea	Randomized double-blind placebo-controlled trial	45 randomized; 44 evaluable (22/22)	Mean 51 vs 50 years	Benign and malignant parotid neoplasms	Partial, superficial, or total parotidectomy; all branches preserved	HB II-IV; mainly one or two facial areas	House-Brackmann	6 months minimum; one permanent case at 18 months	10.1016/j.amjsurg.2008.04.010
Infante-Cossio et al. (2016) [5]	Spain	Prospective randomized controlled trial	79 randomized; 61 had paresis at week 1; moderate-severe subgroup n=45	Mean 48 years (24-81)	Pleomorphic adenoma	Conventional superficial parotidectomy; all facial nerve branches preserved	Postoperative dysfunction measured at week 1	House-Brackmann global and branch scores	1, 3, 6, and 12 months	10.1177/0269215515617309
Boschetti et al. (2022) [17]	Italy	Retrospective case-control study	56 (Kabat 28; control 28)	Mean 53.8 vs 54.0 years	Benign and malignant parotid tumors	Extracapsular dissection, partial, superficial, or total parotidectomy; nerve monitoring used	Severe postoperative FNP: HB IV-V at postoperative day 7	House-Brackmann	7 days; 1, 3, and 6 months	10.3390/diagnostics12030565
Ostrowski et al. (2024) [6]	USA	Case report	1	70 years; male	Oncocytoma	Total parotidectomy; facial nerve dissected, preserved, and responsive to stimulation	Delayed onset postoperative day 12; HB VI	House-Brackmann	10 weeks	10.7759/cureus.55347
Molinari et al. (2024) [4]	Italy	Retrospective multicenter cohort	46	Mean 54 years (17-86)	Benign and malignant lesions	Partial to total/revision parotid surgery; facial nerve anatomically preserved	Any postoperative FNP; 39.1% HB IV-VI at discharge	House-Brackmann and Sunnybrook	2, 6, 12, 18, and 24 months	10.1007/s00405-024-08758-y
Dharwadkar et al. (2025) [9]	India	Case report	1	38 years; male	Warthin tumor	Left superficial parotidectomy; facial nerve preservation not explicitly documented; continuity inferred from the clinical course, not confirmed operatively	Onset postoperative day 5; HB IV	House-Brackmann, facial MMT, strength-duration curve, ICF profile	3 weeks	10.4103/jdmimsu.jdmimsu_184_24
Kuba et al. (2025) [7]	Japan	Case report	1	50 years; female	Basal cell adenoma	Parotid lobectomy; zygomatic and buccal branches preserved; normal stimulation after removal	Initial HB II lower-lip weakness; delayed severe HB V on postoperative day 16	House-Brackmann and electroneurography	4 months after onset; >1 year recurrence surveillance	10.7759/cureus.98700
Alwash (2026) [10]	Iraq	Case report	1	57 years; male	Basal cell adenocarcinoma	Left total parotidectomy; facial nerve preservation not explicitly documented; continuity inferred from the clinical course, not confirmed operatively	HB IV at physiotherapy assessment 4 weeks after surgery	House-Brackmann, Facial Disability Index, MMT, SAQ	6-week treatment and 1-year follow-up	10.1016/j.amjoto.2026.104888

Abbreviations: FDI, Facial Disability Index; HB, House-Brackmann; ICF, International Classification of Functioning, Disability and Health; MMT, manual muscle testing; SAQ, Synkinesis Assessment Questionnaire.

Table S2. Therapeutic protocols and recovery outcomes reported by individual studies

Intervention components, delivery parameters, comparators, study-level outcomes, safety, and interpretive limitations.

Study	Category	Intervention	Protocol / timing / delivery	Comparator	Outcomes measured	Recovery outcomes	Comparative / statistical result	Safety	Key limitation
Roh & Park (2008) [16]	Pharmacological	Oral prednisolone	50 mg/day 5 days, 30 mg/day 3 days, 10 mg/day 2 days; initiated postoperative day 1 or 2 after FNP	Placebo for 10 days	HB recovery at 1, 3, and 6 months	43/44 (98%) fully recovered within 6 months; 37/44 (84%) within 3 months	No difference in recovery rates at any assessed month	One minor prednisolone-related symptom; no major adverse effects	Treatment population and comparator clearly reported
Infante-Cossio et al. (2016) [5]	Rehabilitation / physical therapy	Home mirror facial exercises versus supervised rehabilitation	Control: 10 facial mimic exercises in mirror 5 times/day. Experimental moderate-severe subgroup: ten 45-min weekly supervised sessions with exercises and external/intraoral massage plus home exercises; slight paresis: self-massage and mirror exercises	Active comparator: home exercises	Prevalence, magnitude, duration, and branch recovery using HB-derived scores	No significant between-group differences; 100% probability of complete facial mobility recovery within 12 months when nerve intact	No statistically significant between-group differences (global prevalence p = 0.83; magnitude p = 0.59; recovery p = 0.89); equivalence or non-inferiority was not established	Not specifically reported as treatment-related	Treatment allocation preceded surgery, but therapy was applied to postoperative dysfunction
Boschetti et al. (2022) [17]	Rehabilitation / physical therapy	Early Kabat rehabilitation	Started postoperative day 7; 3 sessions/week for 12-24 weeks; diagonal/spiral PNF movements across upper, intermediate, and lower facial muscle fulcrums	Non-Kabat standard therapy	House-Brackmann at 7 days and 1, 3, 6 months	At 3 months HB grade lower by 0.71 on average; probability of high HB grade reduced by about 13%; 45% of baseline HB V patients reached HB I versus none in control	Significant treatment association at 3 months (p<0.001 linear model; p=0.0168 logistic model); effect less evident by 6 months	Not reported	Retrospective nonrandomized comparison; control refusal may introduce selection bias
Ostrowski et al. (2024) [6]	Pharmacological	High-dose oral prednisone taper	20 mg three times/day for 7 days, then 20 mg twice/day 1 day, 20 mg/day 1 day, 10 mg/day 1 day; second identical course after partial response; eye lubricant	None	House-Brackmann	HB VI at delayed onset to HB II at 10 weeks	Near-complete recovery temporally associated with two steroid courses	Not reported	Single case; spontaneous recovery and inflammatory etiology uncertain
Molinari et al. (2024) [4]	Rehabilitation / physical therapy	Facial neuromuscular retraining	Selective small, slow, symmetric facial movements; external/intraoral massage; mirror feedback; taught by speech therapist then daily home practice; initiated mean 15 days after surgery (range 2-37)	No untreated control; most severe cases also received medical therapy	House-Brackmann and Sunnybrook at baseline, 2, 6, 12, 18, 24 months	Mean Sunnybrook 54 baseline, 76.5 at 2 months, 95.4 at 6 months; complete recovery 72% at 12 months, 85% at 18 months, 89.1% at 24 months; 5/46 HB II at 24 months	Largest improvement within 6 months; statistically significant improvement through 18 months	No rehabilitation-related adverse events reported	82.6% of moderate-severe cases also received prednisone and vitamin B; no control cohort
Dharwadkar et al. (2025) [9]	Rehabilitation / physical therapy	Matrix Rhythm Therapy plus conventional physiotherapy	MaRhyThe 45 min once weekly for 3 weeks, intensity at 10 o'clock position, cervical area and bilateral facial muscles; plus facial exercises, mirror therapy, PNF and functional exercises 10 x 3 repetitions, three times/day	None	House-Brackmann, MMT, strength-duration curve, ICF	HB IV to HB II after 3 weeks; frontalis/orbicularis oculi/oris MMT 2 to 4; improved eye closure, smile and pout	Improvement after combined therapy	No side effects or complications reported; assessment method not described	Single case with multimodal co-intervention
Kuba et al. (2025) [7]	Pharmacological	Intravenous corticosteroid plus antiviral therapy	IV prednisolone sodium succinate 100 mg/day tapered to 40 mg/day over 1 week, then oral prednisolone 30 mg/day tapered to 5 mg every other day over 1 week; IV acyclovir 5 mg/kg every 8 h for 1 week	None	House-Brackmann, MRI, ENoG	HB V on day 16; complete resolution 4 months after onset; no recurrence over >1 year	Complete recovery following combination treatment	Not reported	Single case; no virological confirmation
Alwash (2026) [10]	Rehabilitation / physical therapy	PNF combined with faradic electrical stimulation	12 sessions over 6 weeks (2/week). PNF about 20-30 min/session; faradic current 50 Hz, 12-s surged cycle (6 s on/6 s off), 20 min/session; daily 10-min home exercises; started 4 weeks after surgery	None	House-Brackmann, FDI, MMT, SAQ	HB IV to HB II after 6 weeks; FDI physical 44 to 93 and social 64 to 94; maintained at 1 year; platysma MMT 0 to 3 with residual weakness	Marked functional and patient-reported improvement with persistent cervical-branch deficit	No adverse events, skin irritation, synkinesis, or spasms	Single case; intervention components inseparable; spontaneous recovery possible

Abbreviations: FDI, Facial Disability Index; FNP, facial nerve palsy; HB, House-Brackmann; ICF, International Classification of Functioning, Disability and Health; IV, intravenous; MMT, manual muscle testing; PNF, proprioceptive neuromuscular facilitation; SAQ, Synkinesis Assessment Questionnaire.
